# Distribution of Lactoferrin Is Related with Dynamics of Neutrophils in Bacterial Infected Mice Intestine

**DOI:** 10.3390/molecules25071496

**Published:** 2020-03-25

**Authors:** Li Liang, Zhen-Jie Wang, Guang Ye, Xue-You Tang, Yuan-Yuan Zhang, Jing-Xia Kong, Hua-Hua Du

**Affiliations:** 1 Key Laboratory of Animal Feed and Nutrition of Zhejiang Province, College of Animal Science, Zhejiang University, Hangzhou 310058, China; 21817013@zju.edu.cn (L.L.); 21617013@zju.edu.cn (Z.-J.W.); 3150100240@zju.edu.cn (G.Y.); 21717062@zju.edu.cn (X.-Y.T.); 0017668@zju.edu.cn (Y.-Y.Z.); 2 Department of Social Medicine, School of Medicine, Zhejiang University, Hangzhou 310058, China; 11318261@zju.edu.cn

**Keywords:** Lactoferrin, intestine, antibacterial, inflammatory response, neutrophil

## Abstract

Lactoferrin (Lf) is a conserved iron-binding glycoprotein with antimicrobial activity, which is present in secretions that recover mucosal sites regarded as portals of invaded pathogens. Although numerous studies have focused on exogenous Lf, little is known about its expression of endogenous Lf upon bacterial infection. In this study, we investigated the distribution of Lf in mice intestine during *Escherichia coli (E. coli)* K88 infection. PCR and immunohistology staining showed that mRNA levels of Lf significantly increased in duodenum, ileum and colon, but extremely decreased in jejunum at 8 h and 24 h after infection. Meanwhile, endogenous Lf was mostly located in the lamina propria of intestine villi, while Lf receptor (LfR) was in the crypts. It suggested that endogenous Lf-LfR interaction might not be implicated in the antibacterial process. In addition, it was interesting to find that the infiltration of neutrophils into intestine tissues was changed similarly to Lf expression. It indicated that the variations of Lf expression were rather due to an equilibrium between the recruitment of neutrophils and degranulation of activated neutrophils. Thus, this new knowledge will pave the way to a more effective understanding of the role of Lf in intestinal mucosal immunity.

## 1. Introduction

Lactoferrin (Lf) is a member of the transferrin family of iron binding glycoproteins [1]. It has a wide range of antimicrobial activities, either by depriving pathogens from iron, or by disrupting their plasma membranes through its high cationic charge [2]. Lf is mainly present in most body fluids such as saliva, tears, bile, pancreatic juice, intestinal mucus, seminal fluid and genital secretions [3]. At the mucosa surfaces of the respiratory, urogenital and intestinal tracts, Lf contributes to the primary innate immune defense system and has antimicrobial activity against a variety of pathogens [4]. Thus, Lf has been considered the body’s first line of defense as well as an acute phase protein of innate immunity.

Lf exists in the whole gastrointestinal tract and is considered as a natural compound that can inhibit the growth of pathogenic bacteria [5]. Lf, like lysozyme or defensin, is the first line of defense against bacterial infections. They kill invading bacteria by depleting essential cofactors like free iron, degrading bacterial cell wall components or using other mechanisms that have not been fully elucidated [6]. Furthermore, Lf participates in the regulation of inflammatory response, and plays a key role in maintaining gut homeostasis [7]. Previous studies have shown the expression pattern of Lf in a mouse mammary gland at different lactating stages [8], in the kidney [9] and in epididymis of stallions [10]. Little is known about the endogenous expression pattern of Lf in the intestine. Therefore, this study was designed to identify the endogenous expression of Lf in the murine intestine upon *Escherichia coli* (*E. coli*) K88 infection. We showed that *E. coli* K88 infection elevated the expression of Lf in the villi of duodenum, ileum and colon, but decreased the expression of Lf in the jejunum. Similar infiltration pattern of neutrophils into intestine tissues indicated that the variety of Lf from one intestinal segment to another after infection might be dependent on the number and activation state of neutrophils.

## 2. Results

### 2.1. Establishment of a Murine Model of E. coli K88 Challenge

**Figure 1:**
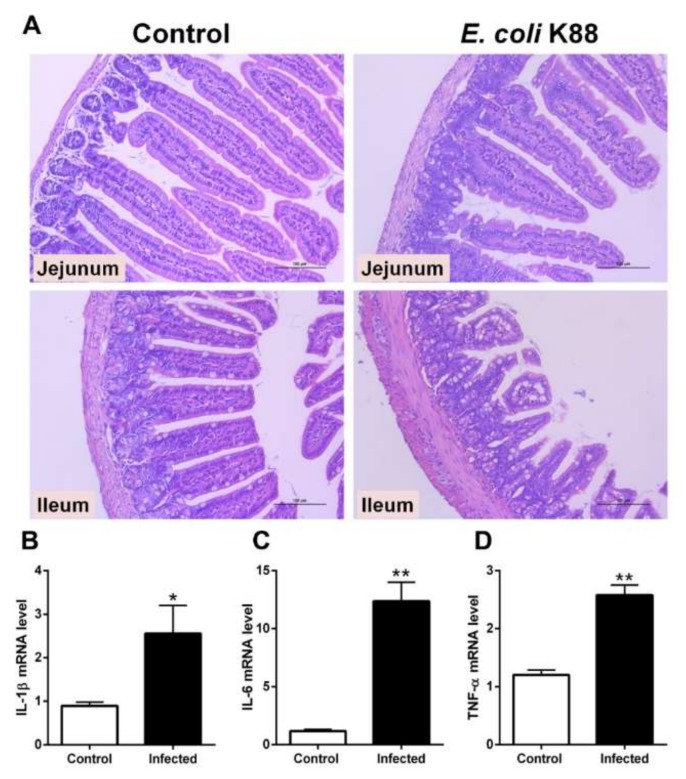
Inflammatory responses to *E. coli* K88 infection. (**A**) Representative H&E-stained section from the jejunum and ileum of mice. Original magnification 400×. In challenged mice, the ileum and jejunum were seriously damaged with discontinuous brush border and short intestinal villi. (**B**–**D**) *E. coli* K88 infection promoted the mRNA expression of IL-1β, IL-6 and TNF-α in mice liver. Data are presented as the mean ± SD, *n* = 6. The values were normalized against the level of GAPDH gene. * Indicates a significant difference (*p* < 0.05) and ** Indicates an extremely significant difference (*p* < 0.01) compared to that of control group.

In order to detect the endogenous expression of Lf, we need to develop a bacterial-challenge model. C57BL/6J mice were intragastrically infected with 10^8^ colony-forming units (CFU) of *Escherichia coli* K88 (*E. coli* K88). After *E. coli* K88 infection, intestinal morphology was damaged and inflammatory responses were stimulated (Figure 1). Hematoxylin and eosin (HE) staining showed that villus height was obviously lower in the jejunum and ileum of challenged mice than in control mice (Figure 1A). After challenge, the edge of villus was unclear and fractured in the ileum. Meanwhile, mRNA expression of inflammatory cytokines including interleukin (IL)-1β, IL-6 and tumor necrosis factor α (TNF-α) were significantly (*p <* 0.05) increased in liver of infected mice (Figure 1B–D). In general, *E. coli* K88 challenge greatly affected villus morphology and the damages were evident.

### 2.2. Expression of Lf in the Intestine of E. coli K88-Infected Mice

**Figure 2:**
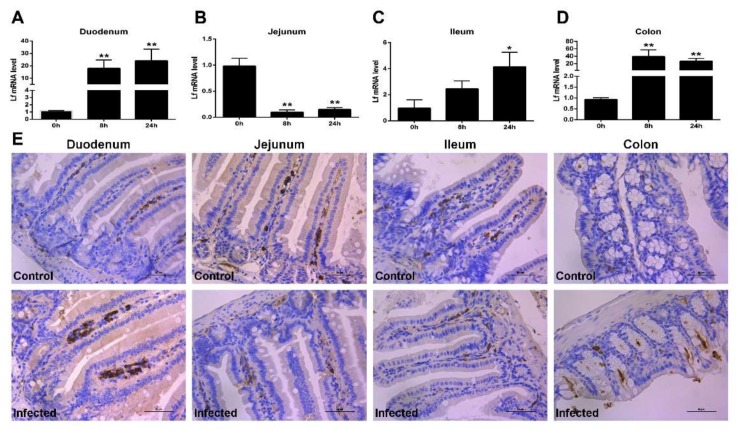
Developmental expression and distribution of Lactoferrin (Lf). (**A**–**D**) *E. coli* K88 infection changed the mRNA expression of Lf in mice intestine including the duodenum, jejunum, ileum and colon with time differences. (**E**) Representative immunohistology-stained section from the duodenum, jejunum, ileum and colon at 0 and 24 h post infection. Original magnification 400×. Formalin-fixed, paraffin-embedded 5-mm cross-sections were stained with a primary Ab to Lf. Data are presented as the mean ± SD, *n* = 6. The values were normalized against the level of GAPDH gene. * Indicates a significant difference (*p* < 0.05) and ** Indicates an extremely significant difference (*p* < 0.01) compared to that of control group or 0 h post challenge.

To determine whether the infection of *E. coli* K88 influences the Lf levels, and whether the levels of Lf differ among different regions of the intestine, samples from four parts of the intestine were studied by real-time PCR (RT-PCR) and Immunohistochemistry (IHC) staining. After *E. coli* K88 infection, the mRNA expression of Lf in all detected intestinal segments of mice were changed distinctly both in 8 h and 24 h post infection (hpi) (Figure 2A–D). The infection induced a remarkably (*p <* 0.05) up-regulated expression of Lf in the duodenum, ileum and colon, but a significantly (*p <* 0.01) down-regulated expression of Lf in the jejunum. At 24 hpi, mRNA levels of Lf of challenged mice were 20-fold higher in the duodenum (Figure 2A) and colon (Figure 2D), and 4-fold in the ileum (Figure 2C) compared with uninfected mice. However, there was a markedly (*p <* 0.01) decreased Lf mRNA expression with 20-fold reduction in the jejunum (Figure 2B) by 8 hpi. In line with changes at the mRNA level, IHC staining analysis showed that Lf protein expression was elevated in the duodenum, ileum and colon at 24 hpi, while expression in the jejunum was reduced after infection (Figure 2E). As the Lf receptor (LfR) plays a key role in the internalization of Lf and facilitates absorption of iron bound to Lf, we also detected the expression of LfR. *E. coli* K88 challenge slightly enhanced the mRNA expression of LfR by 24 hpi in the duodenum, ileum and colon, but markedly (*p <* 0.01) reduced its expression in the jejunum at 8 hpi (Figure S1A–E). The above results showed that Lf was observed in the lamina propria of intestinal villi (Figure 2E), while LfR was mostly localized on the crypts of intestine (Figure S1E).

### 2.3. Distribution of Lf and Neutrophils in the Intestine of E. coli K88-Infected Mice

**Figure 3:**
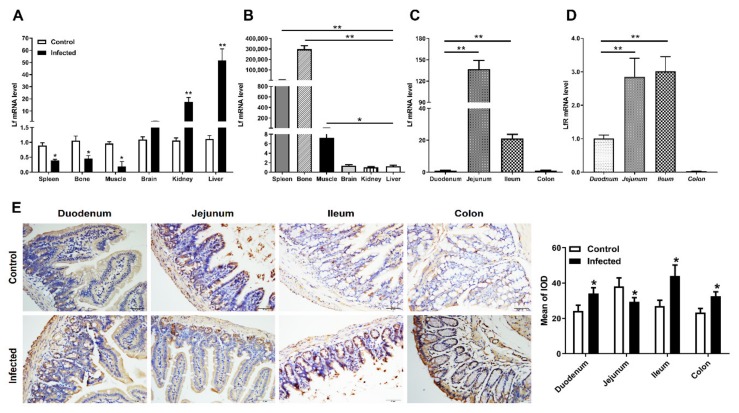
Distribution of Lactoferrin (Lf) and neutrophils in the intestine. (**A**) The changes of mRNA expression of Lf in other organs of challenged mice. (**B**–**D**) The mRNA expression of Lf in tissues and the intestine of wild type mice. (**E**) Representative images of the infiltration of neutrophils in the duodenum, jejunum, ileum and colon at 0 and 24 h post infection. Original magnification 400×. Formalin-fixed, paraffin-embedded 5-mm cross-sections were stained with a primary Ab to myeloperoxidase (MPO). Data are presented as the mean ± SD, *n* = 6. The values were normalized against the level of GAPDH gene. * Indicates a significant difference (*p* < 0.05) and ** Indicates an extremely significant difference (*p* < 0.01) compared to that of control group or 0 h post challenge.

To determine whether the changed Lf mRNA levels upon bacterial infection is not limited to the intestine, other mice organs were collected and studied by RT-PCR. The results showed *E. coli* K88 infection induced an apparently (*p <* 0.05) down-regulated expression of Lf in some tissues (spleen, bone and muscle), but an extremely significant (*p <* 0.01) up-regulation in other tissues (liver and kidney) (Figure 3A). In order to investigate the related values of Lf expression between infected and uninfected mice, we further detected the mRNA levels of Lf in uninfected mice. Lf gene was widely expressed in most tissues of uninfected mice (Figure 3B). Compared with the liver, Lf mRNA was significantly higher expressed in the spleen, bone and muscle. In the intestine of uninfected mice, most mRNA expression of Lf was seen in the jejunum, and the least in the duodenum and colon (Figure 3C), which was distinct from that of LfR (Figure 3D). It indicated that Lf was likely to be modulated to respond to infection, as highly expressed Lf decreased and lowly expressed Lf increased in all detected tissues including the intestine.

Although its origin is not fully understood, Lf seems to be synthesized primarily during neutrophil differentiation and subsequently stored mainly in secondary granules [5]. We further sought to analyze whether Lf expression was mediated through additional modulation of intestinal inflammatory component. In this regard, intestine IHC staining of the myeloperoxidase enzyme (MPO), which is present in neutrophil granules, showed a significant increase (*p <* 0.05) in neutrophil infiltration in the duodenum, ileum and colon after infection (Figure 3E), but a decrease in the jejunum.

## 3. Discussion

Lf is best known for its strong iron binding ability, which is involved in iron transportation and metabolism. Furthermore, Lf is also involved in both innate and adaptive immunities where its modulating effects not only help the host fight against microbes but also protect the host against harmful effects of inflammation [11]. Given the versatile nature of Lf, it is important to determine where Lf is expressed as the first step in understanding its potential role. The present study is the first to demonstrate the expression pattern of Lf in the intestine of mice during *E. coli* K88 infection.

*E. coli* K88 is an extracellular bacterium that produces enterotoxins. Accumulating evidence suggests that *E. coli* K88 causes a high rate of intestinal disorders and diarrhea in newborns and young animals [12]. *E. coli* K88 can easily adhere to and colonize intestinal epithelial cells, destroy epithelial barrier function and increase the risk of intestinal permeability and inflammatory response [13]. *E. coli* infection always promotes the innate immune response in small intestines, including toll-like receptors proteins, proinflammatory cytokines and autophagy [14,15]. In this study, jejunal and ileac intestinal sections demonstrated an obvious atrophy after infection, while inflammatory cytokines were increased in the ileum and jejunum, which aresupposed to activate mucosal immunity. The results indicated that the bacterial challenge model of mice had been successfully established.

In the gut, varieties of antimicrobial substances have been identified in response to infections. Lf is a mediator of both innate and adaptive responses by local and systemic changes in the expression of signaling molecules. The present findings, with the detection of Lf mRNA and protein in the duodenum, jejunum, ileum and colon of the intestine, showed that Lf expression was modulated in the intestine of mice infected by *E. coli* K88, which implies a role of Lf in immune defense. In mucosal fluids, Lf is part of the arsenal of the innate system designed to achieve microbial homeostasis [16]. It might be due to the changes of intestinal flora or the migration of immune cells among different segments of intestine. *E. coli* K88 infection promoted the gene expression of Lf in the duodenum, ileum and colon. We supposed that the increased expression of Lf might be correlated with its function of anti-inflammatory, which is in line with previous report that Lf attenuated the pro-inflammatory response of monocyte-derived macrophages via interaction of the TLR4-specific pathway [17]. Multiple biological activities of Lf depend on its target cells and on the presence of LfR at their surfaces. Our results showed that Lf was observed in the lamina propria of intestinal villi (Figure 2E), while LfR was mostly localized on the crypts of intestine (Figure S1E). The newly synthesized LfR at the bottom of the crypt needs to migrate upward along the villi in order to appear on the apical membrane as a ready-to-be-used receptor for its ligand. Lf binding to LfR may be of importance for iron absorption. Since Lf and LfR were located in the different sites of the intestine, it is hard to say that the endogenous Lf-LfR interaction was implicated in this antibacterial process. It suggests that LfR might be necessary for Lf to enter into intestinal epithelial cells to transport iron, but it might dispensable for Lf to exert its antibacterial or anti-inflammatory activity.

After infection, Lf is released from neutrophils in the blood and inflamed tissues, such as other soluble pattern-recognition receptors of innate immunity [18]. As part of the normal gut inflammatory response, neutrophils are recruited to sites of infection or inflammatory stimuli within minutes, and the response peaks by 24–48 h [19]. Therefore, the strongly punctuated staining pattern in the lamina propria tied well with the presence of Lf-loaded neutrophils. Hence, it is more than probable that the variations of Lf mRNA and protein are rather due to an equilibrium between the recruitment of fresh mature polymorphonuclear neutrophils (PMNs) from blood and the degranulation and further apoptosis of activated PMNs. Such equilibrium between PMN recruitment and Lf degranulation very probably explains why detection of Lf mRNA greatly varies from one intestinal segment to another after infection, depending on the number and activation state of PMNs. The detection of higher Lf RNA levels in naive jejunum, as compared to the other parts of the intestine (Figure 3C) could be explained by the fact that the jejunum is by far the most vascularized and thus contains a greatest number of infiltrated PMNs (Figure 3E). Upon 8h-24h infection, a sound interpretation of the results shown in Figure 1B would be that, unlike the other parts of the intestine, kinetics of degranulation and further apoptosis and/or scavenging of PMNs by macrophages are much faster than recruitment of new blood circulating PMNs. The results presented in the current study may help us to understand the physiological roles of endogenous Lf and determine its therapeutic potential for a variety of intestinal infections.

## 4. Materials and Methods

### 4.1. Animals and Bacteria

Male C57BL/6J mice of 5–6 weeks and 15–18 g body weight was purchased from Shanghai Slack Laboratory Animal Center. Animals were subjected to a 12 h light and dark cycle in a controlled temperature and humidity chamber during the study. They had access to drinking water and food *ad libitum*. Extracellular bacteria *E. coli* K88 was purchased from General Microbiological Culture Collection Center (Beijing, China) and grown in Luria-Bertani (LB). Prior to animal infections, bacteria were cultured overnight in LB medium, washed in a buffer containing 0.1M phosphate-buffered saline (PBS, pH 7.2-7.4) and resuspended in the same buffer to obtain 10^8^ CFU per mL.

### 4.2. Animal Infections

The mice studies were approved by Animal Ethics Committee of Zhejiang University (code number: ZJU2015-447-09). A total of 18 mice were pre-fed for 3 days to suit the experimental environment. They were then randomly grouped into three treatments with six mice per group. All the mice were healthy prior to inoculation. After withdrawing water and feed for 4 h, mice were infected with 0.5 mL of 2 × 10^8^ CFU/mL of *E. coli* K88 intragastrically or treated with PBS twice. During the experiment, the mental state and behavior of the mice were often observed, and the infected mice and the control mice were separately housed. Mice were euthanized by cervical dislocation at 8 or 24 hpi. The duodenum, jejunum, ileum, colon, blood, liver, spleen, kidney, brain and bone marrow of the mice were collected, respectively. Tissue samples were fixed for paraffin sectioning and immunohistochemistry (frozen sections).

### 4.3. RT-PCR

Total RNA isolated from tissues and cells were reversed transcribed using MMLV Reverse Transcriptase (Thermo Fisher Scientific, Shanghai, China). Real-time PCR was performed using Fast Start Universal SYBR Green Master (ROX) (Roche, Shanghai, China) and the ABI 7500 real-time PCR system (ABI StepOnePlus, Applied Biosystems, Foster City, CA, USA). The primer sequences are listed in Table 1. Fold changes were calculated after normalizing the change in expression of the gene of interest to the housekeeping gene GAPDH using the threshold cycle values.

### 4.4. Immunohistochemistry (IHC) Staining

Fresh tissues were fixed in 10% neutral formalin for 24 h, dehydrated, embedded in paraffin and cut into 4 μm thick sections. After dewaxing, the endogenous peroxidase was blocked with 0.3% hydrogen peroxide in methanol solution, and the antigen was repaired with Tris-EDTA buffer (pH 9.0), then blocked with 3% BSA for 30 min, and diluted with the diluted primary antibody (anti-Lf monoclonal Ab, Abcam, 1:100; anti-LfR monoclonal Ab, Abcam, 1:500; anti-Myeloperoxidase (MPO), Servicebio, 1:100) for 1 h. The secondary antibody, which is coupled to horseradish peroxidase, was incubated for 30 min, and finally, the color was developed, the cell nuclei were counterstained with hematoxylin solution, and the sections were observed and photographed with a fluorescence microscope (Leica, Wetzlar, Germany). The mean of integrated optical density (IOD) of MPO was counted using Image-Pro Plus 6.0 (Media Cybernetics, Rockville, MD, USA).

### 4.5. Hematoxylin and Eosin (H&E) Staining

The morphology of duodenal tissues was observed by H&E staining. The duodenum section was fixed at 4% paraformaldehyde, embedded in paraffin, cut into 5 μm blocks and stained with H&E using standard techniques. They were then examined under a DM3000 microscope (Leica, Wetzlar, Germany).

### 4.6. Statistical Analysis

The data were expressed as the mean ± SD of three independent experiments using SPSS 20.0 and GraphPad Prism 6.0 (GraphPad Software, San Diego, CA, USA). The difference between two groups was determined by Student’s *t*-test. One-way ANOVA was used for compared among multiple groups. *P* value < 0.05 was considered statistically significant, while *p* value < 0.01 was considered extremely significant.

## Figures and Tables

**Table 1:** Gene special primers used in the quantitative real-time PCR.

Gene	Forward Primer (5′–3′)	Reverse Primer (5′–3′)
Lf	CCCTACAAACTGCGACCTGT	CACGACTGCTACCGCATAGT
LfR	GTGCAGTGTGGAGACTTTGC	CAATGGAGAAGTCAGGGCCA
IL-1β	AGTTGACGGACCCCAAAAG	TTTGAAGCTGGATGCTCTCAT
IL-6	AGTCCTTCCTACCCCAATTTCC	GGTCTTGGTCCTTAGCCACT
TNF-α	GCTCTTCTGTCTACTGAACTTCGG	ATGATCTGAGTGTGAGGGTCTGG
GAPDH	CCGCATCTTCTTGTGCAGTG	CAAATCCGTTCACACCGACC

## Supplementary Materials

The following are available online, Figure S1: LfR expression in mice intestine.

## References

[1] Baker H.M., Baker E.N. (2012). A structural perspective on lactoferrin function. Biochem. Cell Biol..

[2] Brock J.H. (2012). Lactoferrin--50 years on. Biochem. Cell Biol..

[3] Alexander D.B., Iigo M., Yamauchi K., Suzui M., Tsuda H. (2012). Lactoferrin: An alternative view of its role in human biological fluids. Biochem. Cell Biol..

[4] Jenssen H., Hancock R.E. (2009). Antimicrobial properties of lactoferrin. Biochimie.

[5] Legrand D. (2016). Overview of lactoferrin as a natural immune modulator. J. Pediatr..

[6] Wada Y., Lönnerdal B. (2014). Bioactive peptides derived from human milk proteins--mechanisms of action. J. Nutr. Biochem..

[7] Drago-Serrano M.E., Campos-Rodríguez R., Carrero J.C., de la Garza M. (2017). Lactoferrin: Balancing ups and downs of inflammation due to microbial infections. Int. J. Mol. Sci..

[8] Wang Y., Tu Y., Han F., Xu Z., Wang J. (2005). Developmental gene expression of lactoferrin and effect of dietary iron on gene regulation of lactoferrin in mouse mammary gland. J. Dairy Sci..

[9] åbrink M., Larsson E., Gobl A., Hellman L. (2000). Expression of lactoferrin in the kidney: Implications for innate immunity and iron metabolism. Kidney Int..

[10] Pearl C.A., Roser J.F. (2014). Lactoferrin expression and secretion in the stallion epididymis. Reprod. Biol..

[11] Legrand D. (2012). Lactoferrin, a key molecule in immune and inflammatory processes. Biochem. Cell Biol..

[12] Black R.E. (1990). Epidemiology of travelers’ diarrhea and relative importance of various pathogens. Rev. Infect. Dis..

[13] Ahmed I., Roy B., Khan S., Septer S., Umar S. (2016). Microbiome, metabolome and inflammatory bowel disease. Microorganisms.

[14] Ren W., Chen S., Yin J., Duan J., Li T., Liu G., Feng Z., Tan B., Yin Y., Wu G. (2014). Dietary Dietary arginine supplementation of mice alters the microbial population and activates intestinal innate immunity. J. Nutr..

[15] Tang Y., Li F., Tan B., Liu G., Kong X., Hardwidge P.R., Yin Y. (2014). Enterotoxigenic Escherichia coli infection induces intestinal epithelial cell autophagy. Vet. Microbiol..

[16] Embleton N.D., Berrington J.E., McGuire W., Stewart C.J., Cummings S.P. (2013). Lactoferrin: Antimicrobial activity and therapeutic potential. Semin. Fetal Neonat. M..

[17] Wisgrill L., Wessely I., Spittler A., Förster-Waldl E., Berger A., Sadeghi K. (2018). Human lactoferrin attenuates the proinflammatory response of neonatal monocyte-derived macrophages. Clin. Exp. Immunol..

[18] Mayeur S., Spahis S., Pouliot Y., Levy E. (2016). Lactoferrin, a pleiotropic protein in health and disease. Antioxid. Redox Signal..

[19] Fournier B.M., Parkos C.A. (2012). The role of neutrophils during intestinal inflammation. Mucosal. Immunol..

