# Supplementary material for: Distribution of Lactoferrin Is Related with Dynamics of Neutrophils in Bacterial Infected Mice Intestine
Source: Molecules. 2020 Mar 25;25(7):1496. doi: 10.3390/molecules25071496 (PMC7180821; doi:10.3390/molecules25071496)
Supplement: Supplementary file 1 [file molecules-25-01496-s001.pdf]

Supplementary Materials

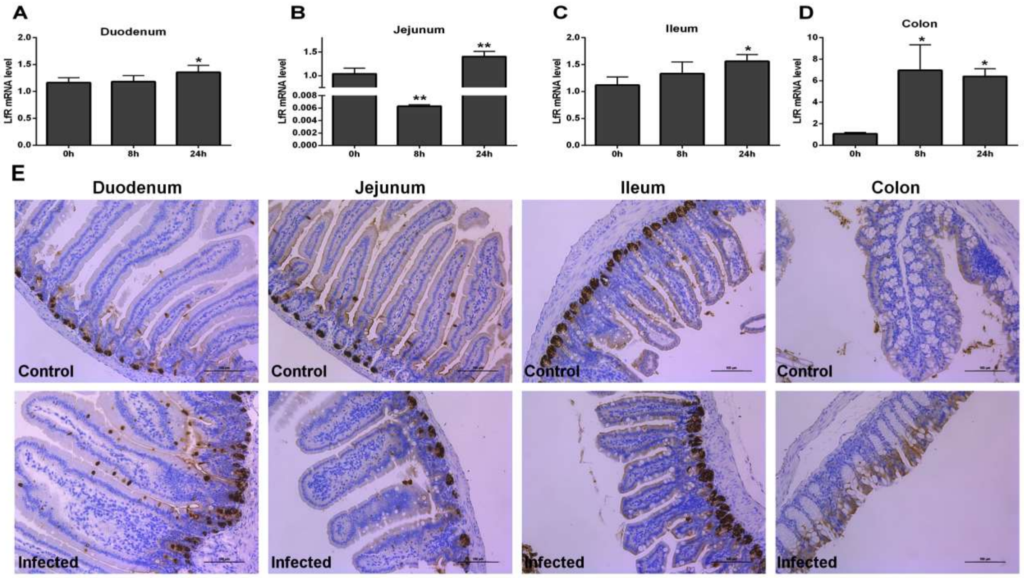

Figure S1. LfR expression in mice intestine. **A-D** *E. coli* K88 infection changed the mRNA expression of lactoferrin in mice intestine including duodenum, jejunum, ileum and colon with time differences. **E** Representative immunohistology-stained section from duodenum, jejunum, ileum and colon. Original magnification 400×. Formalin-fixed, paraffin-embedded 5-mm cross-sections were stained with a primary antibody to LfR. Data are presented as the mean ± SD, n = 6. The values were normalized against the level of GAPDH gene. *Indicates a significant difference ($p < 0.05$) and **Indicate an extreme significant difference ($p < 0.01$) compared to that of control group or 0 h post challenge.
